# Functional Independence Measure score is associated with mortality in critically ill elderly patients admitted to an intermediate care unit

**DOI:** 10.1186/s12877-020-01729-y

**Published:** 2020-09-09

**Authors:** A. D’Andrea, D. Le Peillet, T. Fassier, V. Prendki, V. Trombert, J-L Reny, X. Roux

**Affiliations:** 1 Division of anesthesiology, Département of anesthesiology, Hospital Riviera Chablais, Vaud Valais, Rennaz, Switzerland; 2 Divison of Internal Medecine of the Aged, Departement of Rehabilitation and Geriatrics, Geneva University Hospitals, Geneva, Switzerland; 3 Divison of Internal Medecine of the Aged, Departement of Rehabilitation and Geriatrics, Geneva University Hospitals & Interprofessionnal Simulation Center, Geneva, Switzerland

**Keywords:** Functional status, Intermediate care unit, Mortality, Elderly patients, Functional Independence measure

## Abstract

**Background:**

Age alone is not a robust predictor of mortality in critically ill elderly patients. Chronic health status and functional status before admission could be better predictors. This study aimed to determine whether functional status, assessed using the Functional Independence Measure (FIM), could be an independent predictor of mortality in a geriatric population admitted to an intermediate care unit (IMCU).

**Methods:**

A monocentric, retrospective, observational study of all patients aged ≥75 years old admitted to Geneva University Hospitals’ geriatric IMCU between 01.01.2012 and 31.05.2016. The study’s primary outcome metrics were one-year mortality’s associations with a pre-admission FIM score and other relevant prospectively recorded prognostic variables.

**Results:**

A total of 345 patients were included (56% female, mean age 85 +/− 6.5 years). Mean FIM score was 66 +/− 26. One-year mortality was 57%. Dichotomized low (≤ 63) and high FIM (> 63) scores were associated with one-year mortalities of 68 and 44%, respectively. Logistic regression calculations found an association between pre-admission FIM score and one-year mortality (*p* <  0.0001), including variables usually associated with mortality (e.g., age, sex, comorbidities, mini-mental health state score, renal function). Multivariate survival analysis showed a significant difference between groups, with a hazard ratio of 0.29 (95% CI: 0.13–0.65) for patients with high FIM scores.

**Conclusions:**

In the present study, higher functional status, assessed using the FIM tool before admission to an IMCU, was significantly and independently associated with lower one-year mortality. This opens up perspectives on the potential value of FIM for establishing a finer prognosis and better triage of critically ill older patients.

## Background

As the global population ages, growing numbers of elderly and very elderly patients are being admitted to intensive care units (ICUs) or intermediate care units (IMCUs) [1, 2]. Age has generally been thought to have a strong association with intensive and intermediate care outcomes, with older patients having a poorer prognosis than younger ones [3, 4]. However, several studies have suggested that age alone is not a strong predictor of mortality [5–7]. Indeed, this population’s health status is often difficult to assess because of multiple morbidities and frailty. Better predictors of short and long-term mortality are needed to improve triage decisions [8].

Disease severity scores are well-known tools for evaluating risk of death in the short term. They help clinicians decide whether to admit a patient to an ICU or IMCU, even though their accuracy for very elderly patients is uncertain. The number or severity of a patient’s comorbidities is also often used by clinicians to aid triage decisions on admission to an ICU or IMCU. Frailty seems a promising predictor of mortality. Flatten et al. reported that Clinical Frailty Scale scores were inversely associated with the short-term survival of very elderly patients (≥ 80 years) admitted to an ICU [9]. Indeed, a frailty assessment before admission to an ICU or IMCU may provide more accurate prognostic information than age and comorbidities alone.

The Functional Independence Measure (FIM) provides another functional score, routinely used with geriatric patients to assess their functional status during hospitalization [10]; It is widely used in our hospital for its reliability and reproducibility. FIM scores 18 items of physical, psychological, and social function from 1 to 7, assessing the patient’s level of disability and changes in status in response to rehabilitation or medical intervention. Total scores range from 18 (lowest) to 126 (highest level of function). Tasks evaluated using FIM include sphincter control, transfers, locomotion, communication, social cognition, and the following six self-care activities: grooming, bathing, feeding, upper-body dressing, lower-body dressing, and toileting.

A recent study showed that the systematic admission of critically ill elderly patients to ICUs did not reduce six-month mortality in comparison with usual practice [11]. An effective triage process for elderly patients before ICU or IMCU admission seems necessary and should ideally assess not only comorbidities but also functionality, which is usually difficult to determine, especially during acute organ failure [12].

Although functional status has been identified as a strong predictor of mortality for very elderly patients in ICUs [13], standard hospitalization settings [14, 15], and situations involving bacteremia [14] or pneumonia [15], data are very limited regarding IMCUs. We therefore hypothesized that FIM scores could be important independent predictors for geriatric populations admitted to IMCUs.

## Methods

### Setting

Geneva University Hospitals (HUG) is a 1800-bed Swiss tertiary care institution serving a population of about 500,000 inhabitants; it hosts a 32-bed ICU and a 52-bed IMCU. A 4-bed of geriatric IMCU was created 20 years ago in a dedicated center for elderly patients. This IMCU admits about 400 patients per year, with a mean 4.5-day length of stay [16].

The geriatric IMCU was created for elderly patients who require more intensive care than can be provided in a regular ward: frequent monitoring of vital signs and/or nursing interventions. Patients aged 75 and over with one or more organ failure are triaged by a senior physician on call 24/7, based on the following criteria. Patients are considered eligible for an IMCU when requiring frequent monitoring and/or nursing care were needed but with no criteria for an admission in an intensive care unit (especially if hemodynamic support or mechanical ventilation was required). Discussions regarding cardiopulmonary resuscitation (CPR) or Do-not attempt CPR (DNACPR) orders and decisions to forego-life sustaining treatments (DFLSTs) must be initiated prior to the IMCU admission or within the first 24 h with patients and surrogates or relatives, when possible. They usually do not require invasive monitoring.

### Study design

This monocentric, retrospective, observational study was conducted in the HUG’s geriatric IMCU. Eligible participants were all patients aged 75 years old or more, admitted between 01.01.2012 and 31.05.2016, for any cause. Exclusion criteria were age younger than 75 years, missing data, and an inappropriate IMCU admission, including DFLSTs within the first 24 h. If a patient was admitted to the IMCU several times during the period of observation, only data from the first admission were used.

Data of interest were retrospectively retrieved from patients’ electronic health records (EHRs) including demographic data (age, gender, residential status), clinical data including relevant comorbidities (chronic obstructive pulmonary disease, diabetes, cardiovascular diseases, oncological history, renal failure, neurological diseases), DNACPR orders, and biological data (albumin, urea, creatinine), all recorded before IMCU admission. FIM and mini-mental state (MMS) examination scores were also recorded, when available at the admission of the patients. We included the most recent FIM score available recorded (until 7 days prior admission in IMCU) or the day of admission in the IMCU if not recorded previously. No FIM were retrospectively calculated after the discharge of the patients. Length of stay and mortality in the IMCU stay, and 1 year mortality were also recorded. The national mortality register was consulted if patients were lost to follow-up before 1 year after admission. The date of last patient contact was recorded when one-year survival remained unknown.

### Ethics statement

This study was conducted in accordance with the principles of the Declaration of Helsinki and was approved by the Geneva Ethics Committee (N°2018.00218).

### Outcomes

The primary outcome metric was mortality 1 year after admission to the geriatric IMCU; secondary outcomes were mortality at 28 and 90 days after.

#### Statistical analysis

Baseline characteristics were described using means (standard deviations, SD), medians (interquartile ranges, IQR), and proportions (95% confidence intervals, 95% CI).

#### Univariate analysis

FIM scores were analyzed as continuous, normally distributed variables and expressed as mean ± SD for mortality at day 28, day 90, and day 365; they were compared using Student’s *t*-test. Categorical variables were expressed as numbers and percentages and compared using the chi-squared test or Fisher’s exact test. All *p* values were two-tailed, and *p* < .05 was considered statistically significant.

#### Multivariate analysis

The prognostic relevance of FIM scores was analyzed using a multivariable model. The univariate analysis model considered variables significantly or usually associated with mortality. The backward, multiple logistic regression models used included all the variables yielding a *p* < .2 in the univariate analysis and those considered clinically relevant (age and sex). Possible interactions were tested. The results were summarized as odds ratios with their respective 95% CIs. The Hosmer–Lemeshow goodness-of-fit test was used to evaluate agreement between observed and expected results across all the probability strata of the outcomes of interest (for calibration); *p* > .05 indicated a good fit with the model. FIM scores were dichotomized into low (≤ 63) and high (> 63) scores for a survival analysis over 1 year. This cut-off was previously reported among acute stroke patients to discriminate for patients subject to adverse events (e.g., length of stay) [17].

One-year survival was assessed using the Kaplan–Meier estimate, and a log-rank test tested differences between the two FIM score groups. The Cox proportional hazards regression model was used for the multivariable analysis and results were expressed as a hazard ratio. Statistical analyses were performed using Stata software, version 14.0 IC.

## Results

**Fig. 1:**
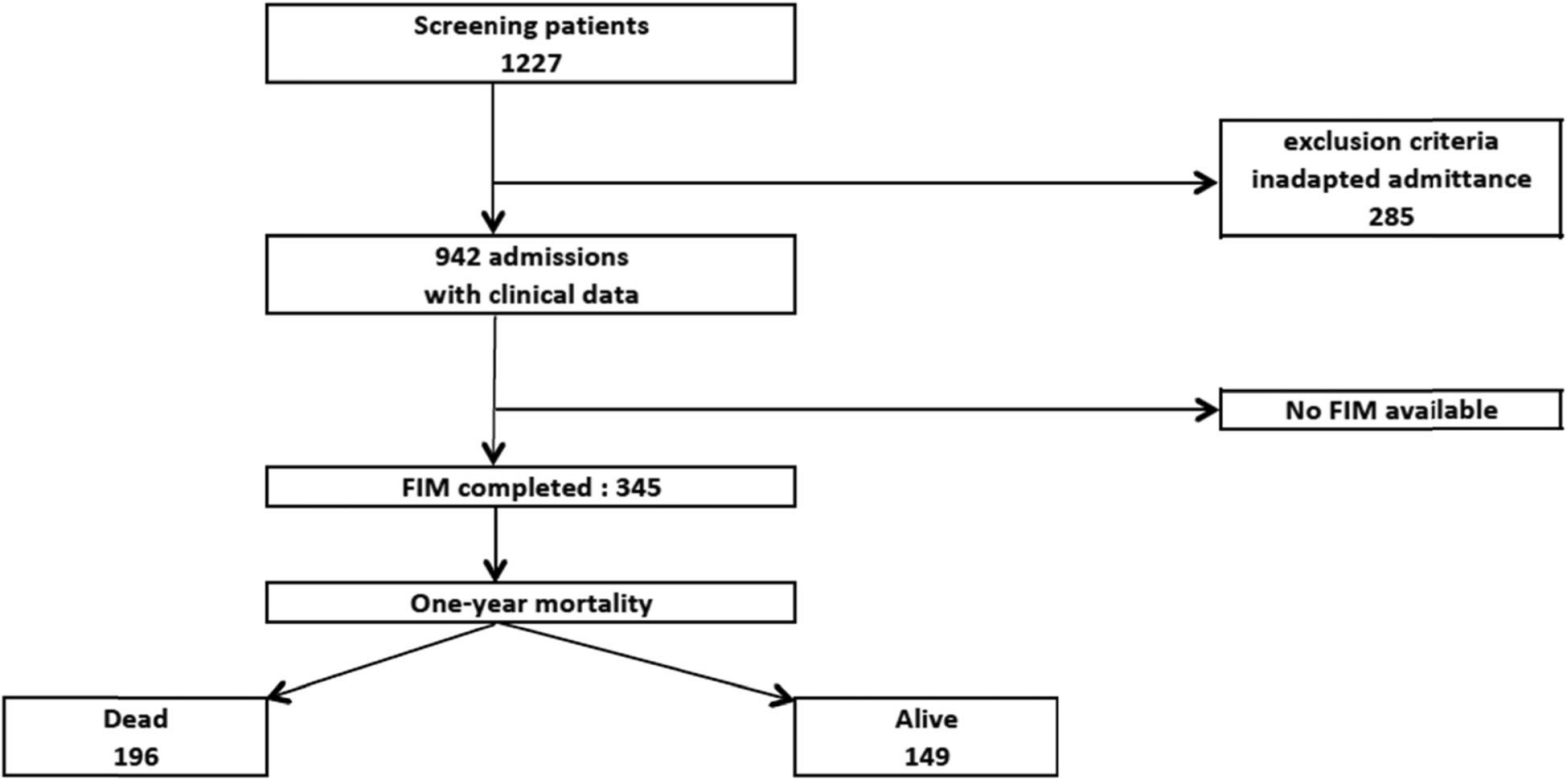
Patient selection flow chart

From 01.01.2012 and 31.05.2016, 1227 patients were admitted in the IMCU and screened for eligibility. Inclusion criteria were met by 345 of 1227 screened patients; 882 were excluded due to loss to follow-up or lack of data (including lack of FIM score) (Fig. 1). Females made up 193 (56%) of those 345 patients, mean age was 85 +/− 6.5 years, and mean FIM score was 66 +/− 26; one-year mortality was 43%; baseline characteristics are summarized in Table 1.

**Table 1:** Study cohort’s baseline characteristics at IMCU admission (using chi-squared test) or Student’s *t*-test)

	All patients *N* = 345	FIM score ≤ 63 *N* = 178	FIM score > 63 *N* = 167	*p*-value
Age, median (IQR), y	85 (79–91)	85 (79–91)	84 (78–91)	
Male, No. (%)	151 (44%)	75 (42)	76 (46)	
Coexisting conditions No. / total No. (%)				
Chronic kidney failure	99 (29%)	46 (26)	53 (32)	0.226
COPD	68 (20%)	34 (19)	34 (20)	0.769
HTA	242 (70%).	126 (71)	116 (70)	0.854
Arrhythmia	125 (36%)	65 (37)	60 (36)	0.909
Diabetes	84 (24%)	43 (24)	41 (25)	0.903
Congestive heart failure	90 (26%)	49 (28)	41 (25)	0.529
Neurological disorders	82 (24%)	47 (26)	35 (21)	0.235
Cancer	59 (17%)	29 (16)	30 (18)	0.680

**Fig. 2:**
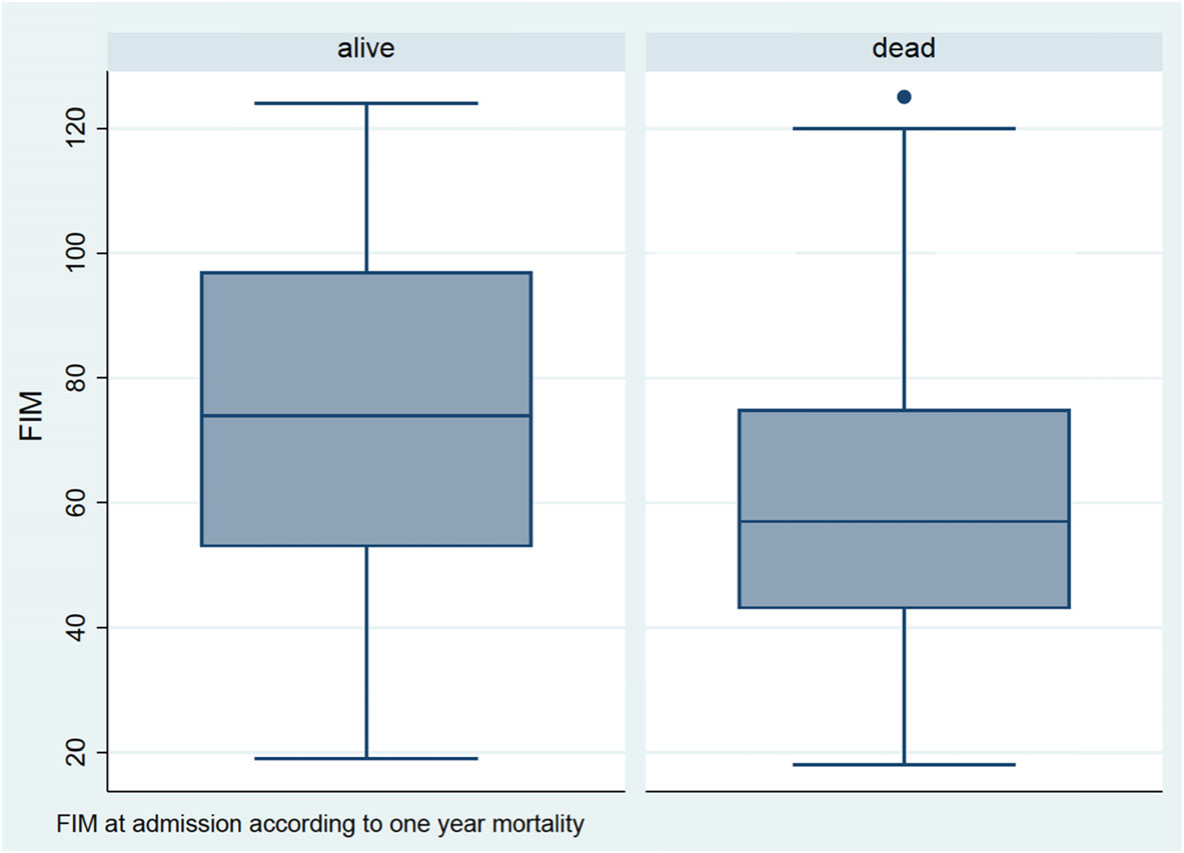
FIM scores at admission. Comparison of FIM scores at admission to the ICU or IMCU between patients alive or dead at 365 days (*p* < 0.0001)

The mean FIM score was 75 among patients alive at 1 year and 60 among patients deceased before then (*p* <  0.0001) (Fig. 2). Regarding other variables, univariate analysis showed a significant difference for MMS, age, albumin, and urea (see Table 1). Low FIM scores were associated with an higher one-year mortality compared with high FIM score (68% vs 44% mortality, *p* <  0.0001). This association was also significant for 28 and 90-day mortalities (35% vs 17%, *p* <  0.0001; 51% vs 27%, *p* <  0.0001, respectively). In the univariate analysis, MMS, urea, albumin, and age appeared to be correlated with one-year mortality (*p* <  0.05) (Table 2).

**Table 2:** Associations between variables at admission and one-year mortality (univariable analysis) using logistic regression

Variable	Odds Ratio	95% Confidence Interval	*p*-value
Age	**1.04**	**1.01–1.08**	**0.01**
Sex	**1.35**	**0.86–2.13**	**0.17**
FIM score	**0.98**	**0.97–0.99**	**< 0.0001**
MMS score	**0.87**	**0.81–0.95**	**0.001**
Cardiopathy^a^	**0.99**	**0.60–1.66**	**0.97**
Cancer	**1.04**	**0.57–1.92**	**0.89**
Diabetes	**0.9**	**0.45–1.80**	**0.74**
Stroke	**0.63**	**0.36–1.13**	**0.09**
COPD	**1.29**	**0.73–2.32**	**0.36**
Chronic Renal Failure	**1.32**	**0.80–2.19**	**0.25**
Urea	**1.09**	**1.05–1.13**	**< 0.0001**
Albumin	**0.90**	**0.86–0.95**	**< 0.0001**

^a^*Chronic heart failure, systolic or diastolic*

After adjustment for other typical predictors of mortality, multivariable analysis found a significant association between FIM scores at admission and one-year mortality (*p* < 0.002). The multivariable model included variables such as sex, age, albumin, urea, stroke, and MMS examination score (Table 3).

**Table 3:** Associations between variables at admission and one-year mortality (multivariable analysis) using Cox regression model. Kaplan–Meier survival estimates for mortality at 1 year after admission to the IMCU with regards to low (blue curve) or high (red curve) FIM scores. FIM scores were dichotomized into low (≤ 63) and high (> 63) FIM score

Variable	Hazard Ratio	95% Confidence Interval	*p*-value
Age	**1.01**	**0.95–1.08**	**0.76**
Sex	**0.94**	**0.41–2.14**	**0.88**
FIM score	**0.97**	**0.96–0.99**	**0.002**
MMS score	**0.95**	**0.88–1.02**	**0.14**
Stroke	**1.27**	**0.59–2.76**	**0.54**
Urea	**1.11**	**1.03–1.19**	**0.005**
Albumin	**0.96**	**0.91–1.02**	**0.16**

**Fig. 3:**
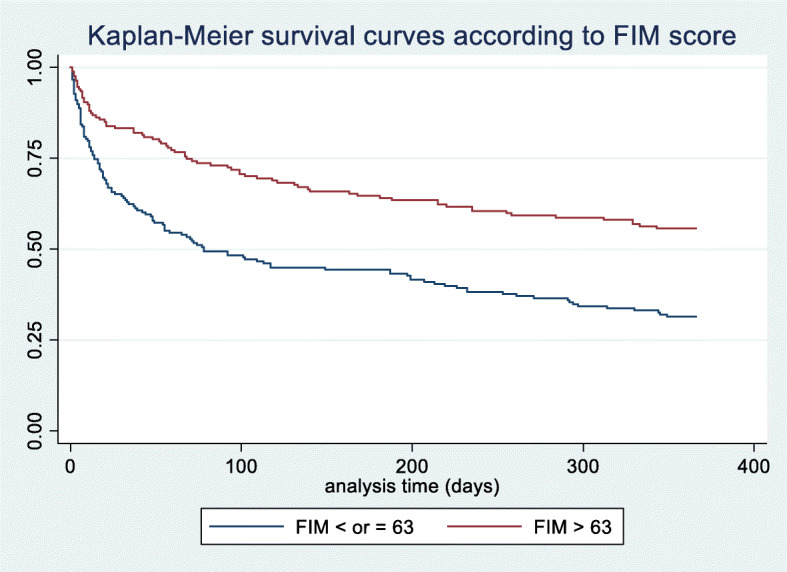
Kaplan–Meier survival estimates according FIM scores

The results of a one-year survival analysis, made using the Kaplan–Meier estimate, compare the low and high FIM score groups in Fig. 3. The difference between the two groups was highly significant (*p* < 0.005). In the Cox regression model, all the potential confounding factors found in the univariate analysis were included (i.e., sex, age, albumin, urea, and MMS examination score). The one-year mortality hazard ratio for low FIM scores was 0.29 (95% CI 0.13–0.65).

## Discussion

The present study showed that elderly patients’ levels of functional independence before admission to an IMCU were strongly associated with one-year mortality, independently of other predictors. Many severity scores already exist, based on physiological status and clinical and biological parameters at the time of care [18]. However, elderly patients are usually under-represented among clinical studies, especially among patients admitted into ICUs. In addition, the basic physiological parameters alter at admission and are thus potentially already markers rather than valid predictors of outcomes: physiological severity scores should be interpreted with caution. Several studies have reported the effects of baseline functional status on the prediction of short- and long-term outcomes [9, 19]. However, there is currently no specific score considering the functional evaluation of elderly patients awaiting a decision on admission to an ICU or IMCU.

The FIM is a score tool which assesses elderly adults’ ability to take care of themselves, and monitors the progress in their rehabilitation. Although other tools for assessing functional status exist, FIM remains easy to use and is commonly used in geriatrics [20]. Developed initially for patients in rehabilitation, FIM was used as a long-term predictor for measuring quality of life among patients suffering from stroke or a neurological disease. However, FIM proved itself to be low-cost, non-invasive, and replicable over time [21]. FIM’s reliability and validity are generally reported to be good, assessing not only neurological impairment but every aspect of the patient’s functional status. The evaluation of elderly patients’ functional and cognitive statuses is key to a discussion about the appropriateness of admitting them to an ICU or IMCU. The lack of a validated prognostic score or scale for elderly populations is a worrying issue as the population ages and in contexts of limited resources and the need to control costs.

To the best of our knowledge, FIM has never been studied as predictor for admitting ICU or IMCU patients. As an easy to use, reproducible instrument, FIM may prove valuable in efforts to improve care and help healthcare professionals select those patients over 75 years old who are most likely to benefit from admission to an IMCU, in addition with other criteria. Indeed, our study showed a high rate of mortality at 1 year after admission to our IMCU (54% mortality), despite the selection criteria for admission, based on discussions with patients, families, general practitioners, and about comorbidities. The IMCU mortality rate obtained at 1 year in our study seems to be comparable to those obtained in other studies among elderly and very elderly patients in ICUs [22]. However, there is great variability in IMCU mortality rates depending on patients’ characteristics at admission. Indeed, medical IMCUs usually exhibit higher mortality than surgical IMCUs, stroke units, and coronary units [23].

Our study found that FIM scores before or at admission to the IMCU were significantly higher among patients still alive at 1 year, in comparison with deceased patients (75 versus 60 respectively). This difference remained significant in the multivariate analysis.

The survival analysis showed better outcomes among patients with higher FIM scores at admission (*p* < .0001). The Cox regression proportional hazard analysis highlighted the independent predictive value of the FIM score for one-year mortality. Thus, having a high FIM score seemed to be both protective and a determinant factor for one-year mortality. Interestingly, the difference was already established and significant at day 28 and day 90, suggesting the predictive value of the FIM score for short-term outcomes.

The risk stratification of patients admitted to ICUs or IMCUs is generally based on a variety of different values such as age, albumin level, or acute disease classification scales such as APACHE or SAPS2. At IMCU admission, illness severity itself is only a short-term mortality predictor, whereas comorbidities seem to be a more robust long-term mortality predictor [23]. The present study’s results indicated that the FIM score could be an independent predictor of one-year mortality among elderly patients admitted into a geriatric IMCU. The comparison of two groups: those whose FIM score was > 63 and those whose FIM was ≤63 found a significant higher mortality rate for low FIM group. This difference was already significant from day 28, emphasizing that a functional geriatric evaluation is essential before IMCU admission. These findings were consistent with previous studies [9, 24].

The present study’s main strength was its homogeneous population of geriatric patients cared for in acute or rehabilitation settings. It had some limitations, however. As a retrospective, monocentric study carried out with a relatively small number of patients, it should not be generalized to other hospitals. Very few of the patients in our geriatric IMCU were admitted post-surgery as they would have been admitted to a post-operative room or an ICU. There was also a potential for biases, especially before admission. Indeed, physicians have often already taken the patient’s functional status into account, whether subjectively or not, before admission to the ICU or IMCU. Orders not to resuscitate or intubate had no significant impact or association with mortality in our study. However, the majority of patients in our study had a limitation of level of care regarding a potential admission in ICU. It would be interesting for any subsequent, prospective, multi-center study to ensure data completeness which might confirm the present results and aid in their generalization. At the end, about two thirds of patients did not have a FIM filled before or the first day of their admission in our unit, with a possible bias. We performed a comparison between patients with FIM filled and no FIM. Except for the MMS score (lower of 2 points in the group with no FIM), we didn’t found any statistically differences (specially regarding age, comorbidity or biological data) (view Additional file 1).

Although many ICUs use different scores to calculate a prognosis for patient survival and decide whether it is worth the costs of admitting them into such a unit [25], it is very interesting to notice that this is not so with geriatric populations: decisions on their admission must also be based on previous functional status rather than merely age or acute illness. Thus, the FIM score could be considered not only as an aid to prognosis but also as a predictor of functional status after hospitalization in an IMCU—two key points of medical decision-making for admittance into such a unit.

It is well known that very elderly populations are extremely different from younger ones, and over 80 years old, the prognostic value of a patient’s chronological age is poor. Instead, the important value seems to be “biological or physiological age”, which is more closely associated with functional status [13]. Decisions such as admission to an IMCU should be based on both an acute diagnosis and previous functional status, and the FIM score may be a useful guide to medical decision-making when IMCUs are crowded. Future studies will be required to prospectively evaluate geriatric populations and the real associations between FIM scores and their cut-off levels and IMCU mortality, the activities of daily living, the instrumental activities of daily living, cognitive impairment, or body mass index [26, 27].

## Conclusion

The Functional Independence Measure (FIM) score seems to be a promising prognostic instrument for assessing the functional status of geriatric patients. The present study showed that pre-admission FIM scores were strongly associated with mortality over a one-year follow-up, with the most significant association being with mortality during the first 90 days after admission.

Using FIM as an indicator of mortality in critically sick elderly patients could be done independently of the other, common, mortality prognosis instruments regularly used in intermediate care units (IMCUs) but which do consider previous functional status. FIM could also potentially be used to support shared decision-making by the internist–geriatrician in charge of the IMCU and the intensivist while taking into account the patient’s values and preferences.

Further prospective studies on the reliability and feasibility of using FIM at IMCU admission are needed. The creation of a dichotomized FIM or integrating FIM into a pre-existing instrument are interesting clinical research hypotheses for the future. Ultimately, a clinical impact study on the usefulness of FIM and other similar relevant instruments is needed to create improved triage tools for critically ill elderly patients who may urgently require a higher level of care.

## Supplementary information

**Additional file 1.** Appendix. Screened cohort’s baseline characteristics at IMCU admission according to FIM availability. Figure 1 with normal distribution of the mains continuous/categorical variable.

## Data Availability

The datasets generated during and/or analysed during the current study are available from the corresponding author on reasonable request.

## Abbreviations

FIM: Functional Independence Measure
IMCU: Intermediate care unit
ICU: Intensive care unit
CPR: Cardiopulmonary resuscitation
MMS: Mini-mental state
DNACPR: Do-not attempt CPR
DFLSTs: Decisions to forego-life sustaining treatments
