# Supplementary material for: Functional Independence Measure score is associated with mortality in critically ill elderly patients admitted to an intermediate care unit
Source: BMC Geriatr. 2020 Sep 9;20:334. doi: 10.1186/s12877-020-01729-y (PMC7488031; doi:10.1186/s12877-020-01729-y)
Supplement: Supplementary file 1 — Additional file 1. Appendix. Screened cohort’s baseline characteristics at IMCU admission according to FIM availability. Figure 1 with normal distribution of the mains continuous/categorical variable. [file 12877_2020_1729_MOESM1_ESM.docx]

**Table I.** Screened cohort’s baseline characteristics at IMCU admission according to FIM availability

|  | FIM available  N = 345 | FIM not available  N = 597 | | *p*-value | |  |
| --- | --- | --- | --- | --- | --- | --- |
| Age, median (IQR), y | 85 (79–91) | 86 (79–93) | | 0.09 | |  |
| Male, No. (%) | 151 (44%) | 228 (38%) | | 0.077 | |  |
| Coexisting conditions  No. / total No. (%) |  |  | |  | |  |
| Chronic kidney failure  COPD  HTA  Arrhythmia  Diabetes  Congestive heart failure  Neurological disorders  Cancer | 99 (29%)  68 (20%)  242 (70%).  125 (36%)  84 (24%)  90 (26%)  82 (24%)  59 (17%) | | 137 (23%)  98 (16%)  403 (68%)  207 (35%)  132 (22%)  151 (25%)  158 (26%)  103 (17%) | | 0.05  0.201  0.365  0.629  0.890  0.788  0.360  0.952 | |
| NTBR | 24 (6.9) | 31 (5.1) | | 0.266 | |  |
| One year Mortality | 198 (57) | 310 (52) | | 0.147 | |  |
| MMS score  Urea  Albumin  stroke | 20.7  11.0  30  67 | 18.6  11.8  30  140 | | 0.0089  0.157  0.747  0.146 | |  |
|  |  |  | |  | |  |

**Figure I** : normal distribution of the mains continuous/categorical variable
